# Clinical pharmacist recommendations in daily interdisciplinary ward rounds at a psychiatric hospital: a retrospective pre-post study on drug-related problems focused in somatic comorbidities

**DOI:** 10.3389/fpsyt.2024.1473832

**Published:** 2024-12-20

**Authors:** Matej Stuhec, Anteja Gorjan Gazdag, Zala Cuk, Robert Oravecz, Borjanka Batinic

**Affiliations:** ^1^ Medical Faculty Maribor, Department of Pharmacology, University of Maribor, Maribor, Slovenia; ^2^ Department of Clinical Pharmacy, Ormoz’s Psychiatric Hospital, Ormoz, Slovenia; ^3^ Psychiatry, Ormoz’s Psychiatric Hospital, Ormoz, Slovenia; ^4^ Faculty of Philosophy, Department of Psychology, University of Belgrade, Belgrade, Serbia; ^5^ Clinic of Psychiatry, University Clinical Centre of Serbia, Belgrade, Serbia

**Keywords:** psychiatry, clinical pharmacy, hospital, ward rounds, drug-related problem

## Abstract

**Objective:**

One potential strategy to address inadequate screening for somatic comorbidities among patients with mental disorders is to integrate a clinical pharmacist into the inpatient team for daily interdisciplinary ward rounds. This approach remains under-researched in psychiatric hospitals. This study aimed to evaluate the impact of a clinical pharmacist on drug-related problems (DRPs) during daily ward rounds within an interdisciplinary team in a psychiatric hospital.

**Methods:**

A retrospective observational pre-post study was conducted at the Ormož Psychiatric Hospital in Slovenia, including patients treated between 2019 and 2020, during which clinical pharmacists offered recommendations during daily ward rounds. The primary outcomes assessed the difference in the total number of DRPs observed at the time of hospital discharge compared to previous stage, as well as the recommendations and their continuation rate after three months. The secondary outcomes evaluated adherence to treatment guidelines.

**Results:**

The study included 186 patients (mean age: 58.1 years, SD=17.0). During ward rounds, 280 recommendations related to DRPs were conducted (1.5 recommendations per patient). Regarding the nature of DRPs, 154 (55.0%) were identified as expressed DRPs, while 127 (45.0%) were deemed potential DRPs. Following pharmacist recommendations, 133 (86.4%) of the expressed DRPs were successfully resolved. The majority of DRPs pertained to treatment effectiveness (N=179, 63.9%), followed by unnecessary treatments (N=86, 30.7%) and patient safety (N=15, 5.4%). Initially, the acceptance rate of recommendations was 88.9% (N=249) at discharge, declining to 63.2% (N=177) three months after discharge. The acceptance rate for somatic conditions at discharge was 87.8% (N=122), declining to 59.0% (N=82) three months after discharge. Adherence to treatment guidelines for somatic comorbidities increased (p < 0.05).

**Conclusions:**

The results indicate that this approach led to fewer DRPs, a high rate of acceptance, and better adherence to treatment guidelines. This is the first retrospective pre-post study in the European Union to include this collaboration in daily rounds at psychiatric hospitals, focusing on somatic comorbidities. However, the study also has significant limitations, such as its non-randomized design and short monitoring period, which should be addressed in future research.

## 1 Introduction

Patients with mental disorders often have many comorbidities, leading to frequent treatment with multiple medications (1, 2). Comorbidities significantly impact mortality, representing a critical issue for pharmacotherapy optimization. Consequently, drug-related problems-DRPs (e.g., drug-drug interactions (DDIs), non-optimal treatment of comorbidities, potentially inappropriate medications (PIMs), irrational polypharmacy) pose a significant challenge in treatment, particularly among older adults (1, 3, 4). In a German cross-sectional study using ambulatory claims data covering 87% of the German population (N = 6.3 million cases and N = 25.2 million in a control group), the three most prevalent somatic comorbid diagnosis groups in patients with depression were other dorsopathies, hypertension, and metabolic disorders. This finding highlights the challenges in treating comorbidities (1). According to a matched-cohort study by Bitter et al., patients with schizophrenia (n = 65,169) had statistically significantly higher all-cause mortality rates than control participants (n = 325,435) (risk ratio = 2.4; P < 0.0001). The most frequent comorbidities were cerebro- and cardiovascular diseases (53.7%), underscoring the necessity of managing comorbidities in daily practice (2). Cardiovascular diseases contribute to approximately a 20% reduction in life expectancy for patients with schizophrenia compared to the general population. Diabetes, cigarette smoking, dyslipidemia, and hypertension are the main risk factors for premature death (5). However, only 25% and 10% of patients initiating antipsychotics are screened for glucose and lipid abnormalities, indicating that most of these patients are not being treated appropriately (6). Numerous studies have demonstrated inadequate medical care for schizophrenia patients, particularly in treating cardiovascular disorders (6, 7). In the Clinical Antipsychotic Trials of Intervention Effectiveness (CATIE) study, the percentages of patients treated for diabetes, hypertension, and dyslipidemia were 30.2%, 62.4%, and 88.0% among patients with confirmed somatic diagnoses in the trial (7). The inadequacy in screening for metabolic problems in real-world clinical practice means that few patients receive the necessary treatment in inpatient and outpatient settings. DRPs are especially prevalent in psychiatric hospitals, as shown in the study published by Soerensen et al. (4). The authors found that the prevalence of PIMs in adults with mental disorders was 59% among adult inpatients, including too high doses (16%), DDIs (36%), and high polypharmacy use (4). These results support the development of interdisciplinary treatment strategies.

One possible interdisciplinary approach to reducing DRPs in psychiatric hospitals (inpatient settings) is to involve a clinical pharmacist within the psychiatric inpatient team in daily activities, such as ward rounds, medication reviews, and educational groups with patients (8). This approach has been well described in primary care settings, including patients with mental disorders (3, 9). The study by Stuhec et al. in Slovenian primary care included 48 patients with mental disorders (average age 79.4 years, SD = 8.13) receiving a total of 558 medications (155 for the treatment of mental disorders). Medications decreased by 9.5% after the clinical pharmacist conducted a medication review. All except one accepted intervention (99.1%) were maintained six months after implementation, significantly decreasing DRPs and improving adherence to treatment guidelines (9). There is limited data on this topic in psychiatric hospitals (8). Most studies come from the UK and USA and are methodologically flawed (10). Clinical pharmacists are usually not part of interdisciplinary medical teams in psychiatric settings within the European Union, and this collaboration is almost completely excluded from research in psychiatric hospitals (8). Only one study in the European Union examined the impact of a clinical pharmacist on DRPs during daily ward rounds (n=224 patients). Psychiatrists accepted 295 (93.7%) of the recommendations. After the recommendations, the number of expressed and potential DRPs decreased in 166 (93.8%) and 129 (93.8%) interventions. Still, it did not research the effects on somatic comorbidities (e.g., hypertension, diabetes, pain). The authors noted that clinical cases in psychiatric hospitals are complex, and collaboration with clinical pharmacists led to fewer DRPs and better treatment guidelines adherence (11).

Such complex cases are common in clinical practice but are not necessarily covered by existing treatment guidelines and randomized controlled trials. Although controlled trials are valuable, they often exclude most patients from clinical practice, so well-designed studies with strong ecological validity are needed to improve the management of DRPs (3). In this context, cohort and pre-post studies are especially needed.

This study aimed to evaluate the impact of a clinical pharmacist on DRPs during daily ward rounds within an interdisciplinary team in a psychiatric hospital. We hypothesized that there would be positive effects on outcomes, such as fewer DRPs.

## 2 Methods

### 2.1 Setting

The Ormož Psychiatric Hospital has 100 beds and provides psychiatric care for Eastern Slovenia, including hospital care, daily hospital, and community psychiatry (11). At this hospital, a clinical pharmacist specialist is a full member of the ward team and performs various pharmaceutical interventions established and legislated in Slovenia (e.g., medication review, daily interventions on the ward, seamless care, interventions during ward rounds) (12). Ward rounds include different healthcare professionals, such as psychiatrists, nurses, social workers, psychologists, and clinical pharmacists (11). At the time of the study, comorbidities were assessed only by hospital psychiatrists and clinical pharmacists. In cases of more complex issues, patients may be referred to a somatic hospital. Ward rounds usually take 5 minutes per patient, and clinical pharmacists participate in all wards once weekly (five different wards). A clinical pharmacist is a specialist in clinical pharmacy, which requires three years of training in Slovenia. From 2023, only clinical pharmacist specialists are allowed to provide clinical work independently on the wards in Slovenia. According to the Pharmaceutical Act, all hospitals in Slovenia must offer clinical pharmacy services (13).

### 2.2 Study design and inclusion/exclusion criteria

A retrospective observational pre-post study conducted on the patients hospitalized at the Ormož Psychiatric Hospital in Slovenia, encompassing patients treated between January 1, 2019, and December 31, 2020, during which clinical pharmacists offered recommendations during daily ward rounds. Clinical pharmacists provided recommendations through conversations with ward psychiatrists and recorded them in the hospital’s electronic system immediately after ward rounds (pre-phase = before recommendations, post-phase = consequences extracted from the hospital electronic system). All recommendations during daily ward rounds were recorded in the hospital’s electronic system in a standardized way (according to the standard operational procedure), which means that all recommendations were recorded in the same way (good data quality). The decision to accept these recommendations was entirely up to the ward psychiatrists, as clinical pharmacists did not have prescribing rights. Clinical pharmacists provided recommendations when they identified a need for medication optimization. These recommendations were brief and recorded in the hospital’s electronic system. Clinical pharmacists documented all recommendations during the study period as part of their routine work.

The study included all adult (>18 years old) patients screened by clinical pharmacists during the study period. Patients of all age categories were included, provided they had at least one mental disorder as defined by the 10th revision of the International Statistical Classification of Diseases and Related Health Problems (ICD-10) (14). Patients with all somatic comorbidities were included. Infectious diseases were excluded due to their short-term treatment and because clinical pharmacists did not collaborate on all antibiotic selections in the hospital during this period. Only patients with somatic comorbidities, including hypertension, heart failure, diabetes, and pain, were included in the further analysis (selection was based on frequencies). Only patients with a complete dataset were included, and each patient was included only once in the study. Only ward-round recommendations provided by a clinical pharmacist were included. This study adhered to the Statement on Strengthening the Reporting of Observational Studies in Epidemiology (STROBE) (15).

### 2.3 Outcomes

The primary outcomes assessed the difference in the total number of DRPs observed at the time of hospital discharge compared to previous stage (before clinical pharmacist recommendations), as well as the recommendations and their continuation rate after three months (percentage). In Slovenia, psychiatrists prescribe medications for each patient for three months after discharge. Therefore, treatment continuation was assessed by comparing prescriptions three months post-discharge with those issued at the time of discharge. DRPs were categorized as either expressed or potential and were linked to treatment efficacy, adverse events, and unnecessary drug treatments. Recommendations were classified into drug discontinuation, initiation, replacement, or dosage/regimen adjustments. DRPs were categorized according to the Slovenian classification of drug-related problems (DRP-SLO-V1) with some modifications (16).

The secondary outcome was adherence to treatment guidelines for somatic comorbidities, including hypertension, heart failure, diabetes, and pain. Different treatment guidelines were used (17–20). When the guidelines provided insufficient data, various studies and summaries of product characteristics were used to determine if the medication use was appropriate. Researchers, who are experts in clinical psychiatry and pharmacy, checked adherence to the guidelines on a case-by-case basis and reached a final consensus for each case.

### 2.4 Data collection and statistics

Data were gathered through retrospective reviews of medical records in the hospital’s electronic system. The two researchers (Z.C. & A.G.G.), who are not clinical pharmacists and were not involved in daily rounds, collected and extracted the data in 2024 after obtaining ethical approval. The researchers included pharmacists and medical specialists (two psychiatrists), who verified the data quality during collection and provided an external review to minimize bias. The somatic comorbidities were grouped based on the ICD-10.The main results were described in numbers using descriptive statistics. The Shapiro-Wilk test checked normality, and the Wilcoxon signed-rank test (non-normally distributed variables) was used to calculate pre-post differences. Researchers set the p-value at 0.05. The sample included all patients for whom the clinical pharmacist made recommendations during the daily rounds.

Data analysis was conducted using the Statistical Package for the Social Sciences (SPSS) 22.0. In 2024, the National Medical Ethics Committee in Slovenia obtained ethical approval (number: 0120-544/2023-2711-6).

## 3 Results

### 3.1 Baseline characteristics

**Figure 1:**
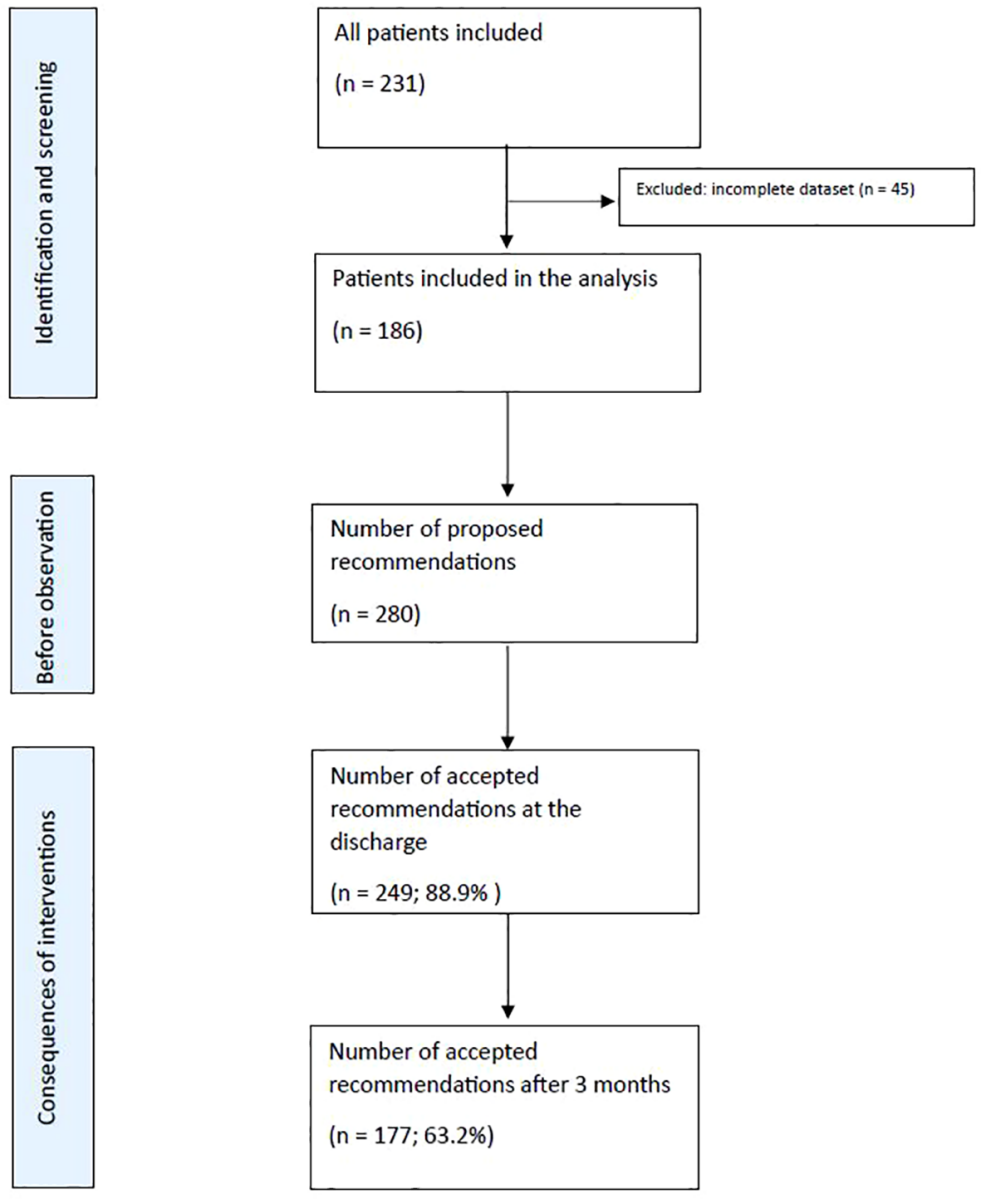
Flowchart for this study.

The study included 186 patients with an average age of 58.1 years (SD=17.0). On average, patients were discharged with 5.6 drugs (median 5, range: 0-16). The gender distribution was nearly evenly split, with 50.5% (N=94) male and 49.5% (N=92) female patients. Results are summarized in the flowchart (**Figure 1**).

### 3.2 Primary outcomes (drug-related problems)

#### 3.2.1 General results

During ward rounds, 280 recommendations related to DRPs were conducted, averaging 1.5 recommendations per patient. Out of these recommendations, 141 (50.4%) were related to mental disorders, and 139 (49.6%) were related to somatic DRPs. Regarding the nature of DRPs, 154 (55.0%) were identified as expressed DRPs, while 127 (45.0%) were deemed potential DRPs. Following pharmacist recommendations, 133 (86.4%) of the expressed DRPs were successfully resolved. The majority of DRPs pertained to treatment effectiveness (N=179, 63.9%), followed by unnecessary treatments (N=86, 30.7%) and patient safety (N=15, 5.4%).

The acceptance rate of recommendations was 88.9% (N=249) at discharge, declining to 63.2% (N=177) three months after discharge.

#### 3.2.2 Results focused on somatic comorbidities

In this study, we focused on somatic comorbidities. The gender distribution of these patients was 43.6% male (N=48) and 56.3% female (N=62). Out of all somatic recommendations (N=139, 49.6% of all recommendations), the majority were related to cardiovascular DRPs (N=75, 54.0%), followed by pain-related DRPs (N=15, 10.8%), gastrointestinal tract-related DRPs (N=8, 5.7%), diabetes-related DRPs (N=7, 5.0%), epilepsy-related DRPs (N=6, 4.3%), and abnormalities in laboratory test results (N=4, 2.9%). Other recommendations (N=24, 17.3%) addressed various DRPs related to different organ systems, such as osteoporosis, tremors, and vertigo, which could not be grouped into smaller categories.

Of all somatic DRPs, 62.6% (N=87) were expressed problems, while 37.4% (N=52) were potential problems. The majority of DRPs in each individual group were expressed problems (average 72.3%), except for epilepsy-related DRPs, where most of the problems were potential (N=5, 83.3%), and gastrointestinal tract DRPs, which were all potential (N=1).

The acceptance rate for somatic conditions at discharge was 87.8% (N=122), declining to 59.0% (N=82) three months after discharge. The acceptance rate of expressed cardiovascular DRPs was 87.7% (N=50); similarly, the acceptance rate of potential DRPs was 83.3% (N=15). **Table 1** provides detailed information on the proposed and accepted recommendations for cardiovascular and diabetes DRPs at discharge and three months after discharge.

**Table 1:** Proposed and accepted recommendations for cardiovascular DRPs and diabetes DRPs at discharge and three months after discharge.

Case number	Problem	Clinical pharmacists recommendations	Acceptance at discharge (yes/no)	Age	Treatment guidelines (yes/no)	Acceptance after three months (yes/no)
1a, 1b	Unstable hypertension	Duloxetin discontinuation was suggested	Yes, 50.0% and No 50.0%	54 and 43	Yes	Yes 100.0%
2a, 2b, 2c, 2d, 2e, 2f, 2g, 2h, 2i, 2j, 2k, 2l, 2m, 2n, 2o, 2p, 2q, 2r, 2s, 2t, 2u, 2v, 2w, 2x, 2y, 2z, 2aa, 2ab	Unsuccessful treatment	Dose adjustment (dose increase or decrease)	Yes, 92.9% and No 7.1%	82 and 78 and 77 and 34 and 44 and 58 and 66 and 70 and 79 and 40 and 81 and 68 and 93 and 72 and 49 and 42 and 29 and 54 and 45 and 54 and 54 and 38 and 42 and 63 and 40 and 84 and 54 and 51	Yes	Yes, 46.4% and No 53.6%
3a, 3b, 3c	Incorrect dosing	Dose correction (perindopril/indapamide 4 mg)	Yes, 66.6% and No 33.3%	46 and 71 and 90	Yes	Yes 100.0%
4a, 4b	Duplication	Discontinuation of medication (combination of beta-blockers)	Yes	81 and 69	Yes	Yes
5a, 5b, 5c, 5d, 5e, 5f, 5g, 5h, 5i, 5j, 5k, 5l	Inappropriate dosing	Dose adjustment (once or twice daily)	Yes 91.7% and No 8.3%	83 and 60 and 75 and 82 and 83 and 69 and 48 and 44 and 59 and 72 and 68 and 51	Yes	Yes, 66.6% and No 33.3%
6	Unsuccessful treatment	Adding ramipril was suggested	No	53	Yes	No
7	Incorrect drug name	Correction	Yes	93	–	Yes
8a, 8b	Time of administration	Time adjustment	Yes 100.0%	42 and 57	Yes	Yes 100.0%
9	Amiodarone in patient with lower blood pressure and sinus rhythm	Switching to bisoprolol was suggested	No	93	Yes	Yes
10	Perindopril and kidney failure	Switching to ramipril was suggested	Yes	66	Yes	No
11a, 11b	Untreated hypertension	Adding perindopril was suggested	Yes, 50.0% and No 50.0%	80 and 43	Yes	No 100.0%
12	Escitalopram and hypotension	Switvhing to venlafaxine was suggested	No	51	Yes	No
13a, 13b, 13c, 13d	RAS inhibitor/diuretic combination and hypotension	Monotherapy was suggested	Yes 100.0%	80 and 81 and 90 and 86	Yes	Yes, 75.0% and No 25.0%
14	Hypotension and antipsychotic therapy	Amisulpride was suggested	Yes	60	Yes	No
15a, 15b, 15c, 15d, 15e, 15f	Hypotension	Discontinuation of medication	Yes 100.0%	52 and 96 and 72 and 49 and 58 and 68	Yes	Yes 66.6% and No 33.3%
16a, 16b	Increased heart rate	Adding bisoprolol was suggested	Yes 100.0%	55 and 49	Yes	Yes 100.0%
17	Telmisartan and increased heart rate	Switching to bisoprolol was suggested	Yes	34	Yes	Yes
18	Metoprolol and anxiety	Switching to bisoprolol was suggested	Yes	82	Yes	Yes
19	Sulpiride and quetiapine combination	Unrecommended combination, discontinuation of one of the medications	Yes	29	Yes	Yes
20	Low heart rate	Discontinuation of medication	Yes	43	Yes	Yes
21	Quetiapine and prolonged QTC interval	Switching to paliperidone was suggested	Yes	86	Yes	No
22	Haloperidol and a history of heart attack	Switching to quetiapine was suggested	Yes	71	Yes	Yes
23	Patient with atrial fibrillation	Adding acetylsalicylic acid was suggested	Yes	77	Yes	No
24	Use of low molecular weight heparin	Dose adjustment	Yes	61	Yes	No
25	Repaglinide as first-line treatment	Switching to metformin was suggested	Yes	69	Yes	Yes
26	Linagliptin restriction	Switching to gliquidone was suggested	Yes	66	Yes	No
27	High blood sugar levels	Adding metformin was suggested	Yes	79	Yes	No
28	Missed medication	Adding medication (insulin) was suggested	Yes	96	Yes	No
29	Hypoglycaemia	Propranolol discontinuation was suggested	Yes	39	Yes	Yes

Pain-related DRPs had a 70.0% (N=7) acceptance rate for expressed problems and an 80.0% (N=4) acceptance rate for potential problems. **Table 2** provides detailed information on the proposed and accepted recommendations for pain-related DRPs at discharge and three months after discharge.

**Table 2:** Proposed and accepted recommendations at discharge and three months after discharge for pain-related DRPs.

Case number	Problem	Clinical pharmacists recommendations	Acceptance at discharge (yes/no)	Age	Treatment guidelines (yes/no)	Acceptance after three months (yes/no)
1a, 1b, 1c	Unsuccessful treatment (neuropathic pain)	Pregabalin dose adjustment	Yes 66.6% and No 33.3%	53, 56 and 65	Yes	Yes 66.6% and No 33.3%
2a, 2b	Unsuccessful treatment (nociceptive pain)	Metamizole dose adjustment	Yes 100.0%	60 and 52	Yes	No 100.0%
3	Unsuccessful treatment (nociceptive pain)	Paracetamol dose adjustment	No	50	Yes	Yes
4	Untreated cold	Adding paracetamol and ascorbic acid was suggested	Yes	87	Yes	–
5	Metamizole for rheumatoid arthritis	Switching to ibuprofen was suggested	Yes	87	Yes	No
6	Unsuccessful treatment (neuropathic pain)	Adding pregabalin was suggested	Yes	47	Yes	Yes
7	Naproxen in patient with cardiovascular comorbidities	Switching to paracetamol was suggested	Yes	42	Yes	No
8	Ibuprofen in patient with acetylsalicylic acid	Switching to paracetamol was suggested	No	50	Yes	No
9	Tizanidine in a subtherapeutic dose	Discontinuation of medication	Yes	77	Yes	Yes
10	Etoricoxib in patient with cardiovascular comorbidities	Switching to metamizole was suggested	No	68	Yes	Yes
11	Etoricoxib in patient with cardiovascular comorbidities	Switching to paracetamol was suggested	Yes	68	Yes	No
12	Paracetamol/tramadol and confusion inpatient	Switching to metamizole was suggested	Yes	76	Yes	No

The pharmacist perceived only potential DRPs related to gastrointestinal problems, with an acceptance rate of 87.5% (N=7). Recommendations related to diabetes were all accepted by 100.0% (N=5). For epilepsy-related problems, the acceptance rates of interventions were 100.0% (N=1) for expressed problems and 80.0% (N=4) for potential problems. DRPs related to abnormalities in laboratory test results were 100.0% (N=4) resolved. Other interventions related to different organ systems addressed 45.8% (N=11) expressed problems, of which 90.9% (N=10) were resolved, and 54.2% (N=13) potential problems, with an acceptance rate of 100.0% (N=13).

### 3.3 Secondary outcomes

Adherence to treatment guidelines for somatic comorbidities increased following the recommendations (p < 0.05). **Table 3** shows the review of somatic disease treatments, including the proportion of accepted recommendations and adherence to treatment guidelines.

**Table 3:** Review of somatic disease treatments, including the proportion of accepted recommendations and adherence to treatment guidelines.

Patients group	Cardiovascular comorbidities	Pain	Diabetes	Other
Number of proposed recommendations	75	15	7	42
Number of accepted recommendations	65	11	7	39
Treatment guidelines adherence (before) % patients	37.3% (N=28)	40.0% (N=6)	14.3% (N=1)	23.8% (N=10)
Treatment guideline adherence (after) % patients	84.0% (N=63)	86.6% (N=13)	100% (N=7)	88.1% (N=37)
Difference	p < 0.05 (0.000)	p < 0.05 (0.008)	p < 0.05 (0.014)	p < 0.05 (0.000)

## 4 Discussion

This is the first retrospective pre-post study in the European Union to include clinical pharmacists’ recommendations in daily rounds at psychiatric hospitals, focusing on somatic comorbidities. These results are crucial in the context of interprofessional collaboration in psychiatric hospitals, where patients often have complex pharmacological needs and significant somatic comorbidities (4).

These results confirmed the positive effects on predefined outcomes as we hypothesized (i.e., fewer DRPs and better adherence to treatment guidelines). Similar results are seen in primary care, where clinical pharmacists’ recommendations in a medication review form have led to better quality pharmacotherapy and a lower number of DRPs in patients with mental disorders (3, 9).

The first important finding is the high acceptance rate by ward psychiatrists (almost 90%) and the numerous DRPs recognized by clinical pharmacists. A high acceptance rate indicates good collaboration between psychiatrists and clinical pharmacists in this hospital. This acceptance rate is much higher than in another study conducted in primary care in Slovenia (50%) and a general hospital in Belgium (60%) (9, 21). In a study conducted in Belgium, the authors assessed a positive impact on the appropriateness of prescribing (21). The high acceptance rate found in our research indicates that daily collaboration on the wards leads to a higher acceptance rate than medication reviews provided in ambulatory settings in primary care (3, 9). This is because clinical pharmacists are full team members on the ward and can provide recommendations directly. Clinical pharmacists’ high number of recognized DRPs (more expressed than potential) suggests a significant clinical impact. This number was higher than in our previous study, which focused on antipsychotics and antibiotics, demonstrating that this collaboration can resolve more DRPs than when psychiatrists work alone (11). The results show that clinical pharmacists possess important clinical competencies and could represent a significant step towards better quality pharmacotherapy for these patients.

The second important finding is the positive impact of reducing the number of DRPs after clinical pharmacist recommendations, with most accepted recommendations being continued. This indicates that clinical pharmacists’ recommendations lead to fewer DRPs in a psychiatric hospital. Clinical pharmacists provided recommendations equally on somatic comorbidities and mental disorders, demonstrating that they identified additional issues not addressed by psychiatrists alone. Patients with mental disorders frequently have somatic comorbidities, and few of them are screened for these conditions (6, 7). Pharmacists identified most DRPs in treatment effectiveness, meaning they recognized issues in clinical outcomes. This was confirmed in another study conducted in Slovenian nursing homes, where a positive impact on the quality of life was observed in patients with mental disorders (22). Although our study did not measure quality of life, the high continuation rate shows that patients remained on the proposed medications. Many recommendations involved initiating medication for untreated conditions (e.g., hypertension), which represented the highest cost-effectiveness ratio among the pharmacist’s recommendations, as demonstrated in studies, including those from Veterans Affairs (23). However, it is commonly recognized that cost-effectiveness data must be interpreted cautiously due to limited comparability between different healthcare systems.

The third important finding is better adherence to treatment guidelines. The existing treatment guidelines supported almost all recommendations, and treatment guidelines adherence improved significantly. Cardiovascular disorders are one of the main reasons for death in patients with mental disorders, and therefore, appropriate pharmacotherapy, including treatment of comorbidities, is needed (2, 5). They are also the leading cause of death globally, resulting in 17.9 million deaths in 2015 (24). Our results align with a study in two clozapine clinics, where clinical pharmacists’ recommendations led to better outcomes focused on comorbidities (weight, glucose level, and lipids) in patients with schizophrenia treated with clozapine. Results show that regular medication reviews by clinical pharmacists improved physical health monitoring for patients receiving clozapine (25). In a cluster randomized study from the US, which included 335 hypertensive patients with comorbidities without mental disorders, the pharmacist-intervention group had a significantly greater mean reduction in systolic blood pressure compared with usual care at 9 months (8.64 mm Hg; 95% confidence interval [CI] -12.8 to -4.49, p<0.001). This demonstrates that collaborative care, including clinical pharmacists, was effective in treating hypertension (26). In our study, clinical pharmacists proposed most of the recommendations for dose adjustments (e.g., tapering and titration) and medication initiation for newly recognized hypertension (e.g., ramipril) or unsuccessfully treated hypertension (new medication). These results align with guidelines, indicating that this collaboration significantly improved adherence to treatment guidelines (18). Most of the patients had systolic blood pressure under 150-160 mmHg and/or were elderly, and therefore clinical pharmacists recommended titration first. Treatment with two drugs is recommended for patients with a systolic blood pressure >20 mmHg and/or a diastolic blood pressure >10 mmHg above the goals, as well as for those with high cardiovascular risk (27).

Similar results were observed in heart failure treatment, where recommendations significantly improved adherence to treatment guidelines (17). More patients were treated with higher doses of beta-blockers than before (over 80% acceptance rate). Beta-blockers are a key medication group in heart failure treatment due to their survival benefits, and therefore, titration to the maximum tolerated dose is recommended (17, 28). Clinical pharmacists recommended titration of beta-blockers to the higher doses, which could potentially impact lower mortality rates (28). For diabetes treatment, recommendations were mostly associated with initiating metformin or switching to metformin, which is also in line with guidelines (29). Results also showed pain treatment guidelines adherence. Pain is often present in patients with mental disorders (30). The prevalence of pain is averaged at 65% in depressed patients across a pooled analysis of multiple studies (30). On the other side, an occurrence of pain could lead to mental disorders, which was shown in the prospective cohort study in Sweden (n=504,365), where the incidence rate ratio for developing mental disorders after pain was 2.18 (95% CI = 2.14-2.22) compared to without pain (31). The results of our study showed positive outcomes, particularly in the treatment of nociceptive pain. Pharmacists also recommended discontinuing etoricoxib in a patient with unstable hypertension (contraindication). On the other hand, pharmacists recommended initiating paracetamol and metamizole for the treatment of nociceptive pain. Both medications are also recommended for elderly patients (20). This suggests that clinical pharmacists can successfully manage patients with mental disorders and pain comorbidities. Similar results have been observed in primary care patients with mental disorders (9). Results shows that clinical pharmacists’ recommendations led to better treatment guidelines adherence also in other somatic comorbidities (e.g., GIT & abnormalities in laboratory tests results). In the GIT field, clinical pharmacists recommended dose adjustment or discontinuation of proton pump inhibitors, which are often used as a long-term treatment without an approved indication. These results are in line with those seen in primary care, where clinical pharmacists’ recommendations reduced the number of patients treated with proton pump inhibitors without an approved long-term indication (32).

But on the other hand, a study has many practical implications, especially clinical relevance. Results show the importance of such collaboration in clinical practice in a psychiatric hospital, which is not the case in each country (8). Results show that clinical pharmacists are important members of the healthcare team on the ward and reduce the number of DRPs. They also show that psychiatrists and clinical pharmacists could recognize and solve more DRPs together. The unanswered questions in this study present opportunities for further research through a long-term study, including the cost-effectiveness of this collaboration, reasons for non-acceptance by psychiatrists and general practitioners, and the impact on quality of life. Additionally, this study could be replicated in more psychiatric hospitals as a prospective study.

### 4.1 Limitations

Despite these positive results, this study also has some significant limitations, which should be mentioned here. Clinical outcomes were not measured with scales. This was not done because a clinical pharmacist screened the heterogeneous population. The study was also not a randomized controlled trial. The study was not prospective, which may result in higher data loss and limits the ability to assess the data more comprehensively. The study also included patients with different comorbidities, which reduced homogeneity and increased the selection bias. The study did not investigate the psychiatric diagnoses in relation to clinical pharmacist recommendations, which could be a point of interest for future studies. Additionally, the clinical pharmacists in this study did not have prescribing rights. Since this was a retrospective short-term study, we did not investigate why psychiatrists and general practitioners later did not accept some recommendations. The study did not investigate the reasons why pharmacotherapy was not continued during the three-month post-discharge period. One of the reasons could be the lack of medication reconciliation by clinical pharmacists at hospital discharge, which was not available in Slovenian hospitals at that time but significantly contributes to fewer DRPs at discharge (12).

## 5 Conclusion

The results of this study demonstrate a positive impact of clinical pharmacist recommendations during daily ward rounds in a psychiatric hospital, including a reduction in DRPs and improved adherence to treatment guidelines. Most recommendations remained unchanged three months after their introduction. Further studies are needed on this topic, mainly prospective randomized studies, to establish the relationship between clinical pharmacists’ recommendations and outcomes in daily practice.

## Data Availability

The original contributions presented in the study are included in the article/Supplementary Material. Further inquiries can be directed to the corresponding author.

## References

[1] Steffen A Nübel J Jacobi F Bätzing J Holstiege J. Mental and somatic comorbidity of depression: a comprehensive cross-sectional analysis of 202 diagnosis groups using German nationwide ambulatory claims data. BMC Psychiatry. (2020) 20:142. doi: 10.1186/s12888-020-02546-8 32228541 PMC7106695

[2] Bitter I Czobor P Borsi A Fehér L Nagy BZ Bacskai M. Mortality and the relationship of somatic comorbidities to mortality in schizophrenia. A nationwide matched-cohort study. Eur Psychiatry. (2017) 45:97–103. doi: 10.1016/j.eurpsy.2017.05.022 28753464

[3] Stuhec M. Antipsychotic treatment in elderly patients on polypharmacy with schizophrenia. Curr Opin Psychiatry. (2022) 35:332–7. doi: 10.1097/YCO.0000000000000808 35788124

[4] Soerensen AL Nielsen LP Poulsen BK Lisby M Mainz J. Potentially inappropriate prescriptions in patients admitted to a psychiatric hospital. Nord J Psychiatry. (2016) 70:365–73. doi: 10.3109/08039488.2015 26824679

[5] Oh J Nam H Park S Chae JH Kim TS. Decreased cardiovascular death in schizophrenia patients treated with antipsychotics: A Korean national cohort study. Schizophr Res. (2021) 228:417–24. doi: 10.1016/j.schres.2021.01.006 33556675

[6] Farley JF Hansen RA Yu-Isenberg KS Maciejewski ML. Antipsychotic adherence and its correlation to health outcomes for chronic comorbid conditions. Prim Care Companion CNS Disord. (2012) 14:PCC.11m01324. doi: 10.4088/PCC.11m01324 23106028 PMC3466037

[7] Lieberman JA Stroup TS McEvoy JP Swartz MS Rosenheck RA Perkins DO. Effectiveness of antipsychotic drugs in patients with chronic schizophrenia. N Engl J Med. (2005) 353:1209–23. doi: 10.1056/NEJMoa051688 16172203

[8] Stuhec M Hahn M Taskova I Bayraktar I Fitzgerald I Molitschnig L. Clinical pharmacy services in mental health in Europe: a commentary paper of the European Society of Clinical Pharmacy Special Interest Group on Mental Health. Int J Clin Pharm. (2023) 45:1286–92. doi: 10.1007/s11096-023-01643-4 PMC10600282 37755642

[9] Stuhec M Lah L. Clinical pharmacist interventions in elderly patients with mental disorders in primary care focused on psychotropics: a retrospective pre-post observational study. Ther Adv Psychopharmacol. (2021) 11:20451253211011007. doi: 10.1177/20451253211011007 34025980 PMC8072848

[10] Werremeyer A Bostwick J Cobb C Moore TD Park SH Price C. Impact of pharmacists on outcomes for patients with psychiatric or neurologic disorders. Ment Health Clin. (2020) 10:358–80. doi: 10.9740/mhc.2020.11.358 PMC7653731 33224694

[11] Stuhec M Tement V. Positive evidence for clinical pharmacist interventions during interdisciplinary rounding at a psychiatric hospital. Sci Rep. (2021) 11:13641. doi: 10.1038/s41598-021-92909-2 34211019 PMC8249606

[12] Stuhec M Batinic B. Clinical pharmacist interventions in the transition of care in a mental health hospital: case reports focused on the medication reconciliation process. Front Psychiatry. (2023) 14:1263464. doi: 10.3389/fpsyt.2023.1263464 38205081 PMC10777203

[13] Urbańczyk K Guntschnig S Antoniadis V Falamic S Kovacevic T Kurczewska-Michalak M. Recommendations for wider adoption of clinical pharmacy in Central and Eastern Europe in order to optimise pharmacotherapy and improve patient outcomes. Front Pharmacol. (2023) 14:1244151. doi: 10.3389/fphar.2023.1244151 37601045 PMC10433912

[14] 10th revision of the International Statistical Classification of Diseases and Related Health Problems (ICD), a medical classification list by the World Health Organization (WHO) . Available online at: https://www.who.int/classifications/icd/en/ (Accessed June 25, 2024).

[15] von Elm E Altman DG Egger M Pocock SJ Gøtzsche PC Vandenbroucke JP. The Strengthening the Reporting of Observational Studies in Epidemiology (STROBE) statement: guidelines for reporting observational studies. J Clin Epidemiol. (2008) 61:344–9. doi: 10.1016/j.jclinepi.2007.11.008 18313558

[16] Horvat N Kos M. Development and validation of the Slovenian drug-related problem classification system based on the PCNE classification V 6.2. Int J Clin Pharm. (2016) 38:950–9. doi: 10.1007/s11096-016-0320-7 27255777

[17] Authors/Task Force Members McDonagh TA Metra M Adamo M Gardner RS ESC Scientific Document Group. 2021 ESC Guidelines for the diagnosis and treatment of acute and chronic heart failure: Developed by the Task Force for the diagnosis and treatment of acute and chronic heart failure of the European Society of Cardiology (ESC). With the special contribution of the Heart Failure Association (HFA) of the ESC. Eur J Heart Fail. (2022) 24:4–131. doi: 10.1002/ejhf.2333 35083827

[18] Mancia G Kreutz R Brunström M Burnier M Grassi G Januszewicz A. 2023 ESH Guidelines for the management of arterial hypertension The Task Force for the management of arterial hypertension of the European Society of Hypertension: Endorsed by the International Society of Hypertension (ISH) and the European Renal Association (ERA). J Hypertens. (2023) 41:1874–2071. doi: 10.1097/HJH.0000000000003480 37345492

[19] Finnerup NB Attal N Haroutounian S McNicol E Baron R Dworkin RH. Pharmacotherapy for neuropathic pain in adults: a systematic review and meta-analysis. Lancet Neurol. (2015) 14:162–73. doi: 10.1016/S1474-4422(14)70251-0 PMC4493167 25575710

[20] Mann NK Mathes T Sönnichsen A Pieper D Klager E Moussa M. Potentially inadequate medications in the elderly: PRISCUS 2.0. Dtsch Arztebl Int. (2023) 120:3–10. doi: 10.3238/arztebl.m2022.0377 36507719 PMC10035347

[21] Stuhec M Zorjan K. Clinical pharmacist interventions in ambulatory psychogeriatric patients with excessive polypharmacy. Sci Rep. (2022) 12:11387. doi: 10.1038/s41598-022-15657-x 35794225 PMC9259566

[22] Stuhec M Bratović N Mrhar A. Impact of clinical pharmacist’s interventions on pharmacotherapy management in elderly patients on polypharmacy with mental health problems including quality of life: A prospective non-randomized study. Sci Rep. (2019) 9:16856. doi: 10.1038/s41598-019-53057-w 31728029 PMC6856189

[23] Lee AJ Boro MS Knapp KK Meier JL Korman NE. Clinical and economic outcomes of pharmacist recommendations in a Veterans Affairs medical center. Am J Health Syst Pharm. (2002) 59:2070–7. doi: 10.1093/ajhp/59.21.2070 12434719

[24] Wang H Naghavi M Allen C Barber RM Bhutta ZA Carter A. Global, regional, and national life expectancy, all-cause mortality, and cause-specific mortality for 249 causes of death, 1980-2015: a systematic analysis for the Global Burden of Disease Study 2015. Lancet. (2016) 388:1459–544. doi: 10.1016/S0140-6736(16)31012-1 PMC5388903 27733281

[25] Spann G Austin L King E. Pharmacists in clozapine clinics improving physical health monitoring. Ment Health Clin. (2022) 12:193–98. doi: 10.9740/mhc.2022.06.193 PMC9190272 35801163

[26] Anderegg MD Gums TH Uribe L MacLaughlin EJ Hoehns J Bazaldua OV. Pharmacist intervention for blood pressure control in patients with diabetes and/or chronic kidney disease. Pharmacotherapy. (2018) 38:309–18. doi: 10.1002/phar.2083 PMC5867244 29331037

[27] Guerrero-García C Rubio-Guerra AF. Combination therapy in the treatment of hypertension. Drugs Context. (2018) 7:212531. doi: 10.7573/dic.212531 29899755 PMC5992964

[28] Ajam T Ajam S Devaraj S Fudim M Kamalesh M. Effect on mortality of higher versus lower β-blocker (Metoprolol succinate or carvedilol) dose in patients with heart failure. Am J Cardiol. (2018) 122:994–8. doi: 10.1016/j.amjcard.2018.05.038 30049457

[29] ElSayed NA Aleppo G Aroda VR Bannuru RR Brown FM Bruemmer D. 9. Pharmacologic approaches to glycemic treatment: standards of care in diabetes-2023. Diabetes Care. (2023) 46:S140–57. doi: 10.2337/dc23-S009 PMC9810476 36507650

[30] Li JX. Pain and depression comorbidity: a preclinical perspective. Behav Brain Res. (2015) 276:92–8. doi: 10.1016/j.bbr.2014.04.042 PMC4216773 24797835

[31] Bondesson E Larrosa Pardo F Stigmar K Ringqvist Å Petersson IF Jöud A. Comorbidity between pain and mental illness - Evidence of a bidirectional relationship. Eur J Pain. (2018) 22:1304–11. doi: 10.1002/ejp.1218 29577509

[32] Bundeff AW Zaiken K. Impact of clinical pharmacists’ recommendations on a proton pump inhibitor taper protocol in an ambulatory care practice. J Manag Care Pharm. (2013) 19:325–33. doi: 10.18553/jmcp.2013.19.4.325 PMC10438075 23627578

